# Clinical evaluation of autoantibodies to a novel PM/Scl peptide antigen

**DOI:** 10.1186/ar1729

**Published:** 2005-04-01

**Authors:** Michael Mahler, Reinout Raijmakers, Cornelia Dähnrich, Martin Blüthner, Marvin J Fritzler

**Affiliations:** 1 Dr Fooke Laboratorien GmbH, Neuss, Germany; 2 Radboud University Nijmegen, The Netherlands; 3 Euroimmun GmbH, Lübeck, Germany; 4 Labor Seelig und Kollegen, Karlsruhe, Germany; 5 Faculty of Medicine, University of Calgary, Canada

## Abstract

Anti-PM/Scl antibodies represent a specific serological marker for a subset of patients with scleroderma (Scl) and polymyositis (PM), and especially with the PM/Scl overlap syndrome (PM/Scl). Anti-PM/Scl reactivity is found in 24% of PM/Scl patients and is found in 3–10% of Scl and PM patients. The PM/Scl autoantigen complex comprises 11–16 different polypeptides. Many of those proteins can serve as targets of the anti-PM/Scl B-cell response, but most frequently the PM/Scl-100 and PM/Scl-75 polypeptides are targeted. In the present study we investigated the clinical relevance of a major alpha helical PM/Scl-100 epitope (PM1-α) using a newly developed peptide-based immunoassay and compared the immunological properties of this peptide with native and recombinant PM/Scl antigens. In a technical comparison, we showed that an ELISA based on the PM1-α peptide is more sensitive than common techniques to detect anti-PM/Scl antibodies such as immunoblot, indirect immunofluorescence on HEp-2 cells and ELISA with recombinant PM/Scl polypeptides. We found no statistical evidence of a positive association between anti-PM1-α and other antibodies, with the exception of known PM/Scl components. In our cohort a negative correlation could be found with anti-Scl-70 (topoisomerase I), anti-Jo-1 (histidyl tRNA synthetase) and anti-centromere proteins. In a multicenter evaluation we demonstrated that the PM1-α peptide represents a sensitive and reliable substrate for the detection of a subclass of anti-PM/Scl antibodies. In total, 22/40 (55%) PM/Scl patients, 27/205 (13.2%) Scl patients and 3/40 (7.5%) PM patients, but only 5/288 (1.7%) unrelated controls, tested positive for the anti-PM1-α peptide antibodies. These data indicate that anti-PM1-α antibodies appear to be exclusively present in sera from PM/Scl patients, from Scl patients and, to a lesser extent, from PM patients. The anti-PM1-α ELISA thus offers a new serological marker to diagnose and discriminate different systemic autoimmune disorders.

## Introduction

Systemic autoimmune diseases such as scleroderma (Scl), polymyositis (PM), rheumatoid arthritis, systemic lupus erythematosus (SLE) and mixed connective tissue disease are characterized by the occurrence of circulating antibodies to defined intracellular targets [1]. Some of these autoantibodies represent useful diagnostic markers for a variety of systemic autoimmune diseases [1,2].

Antibodies targeting the PM/Scl complex serve as a marker for the PM/Scl overlap syndrome, where they are found in 24% of sera, but they are also seen in 8% of PM patients and in 3% of Scl patients [3-6]. The PM/Scl complex was identified as the human counterpart of the yeast exosome and consists of 11–16 polypeptides with molecular masses ranging from 20 to 110 kDa [7-11]. PM/Scl-100, the human equivalent of the yeast Rrp6p, has been cloned by two independent groups and its key function during the 5.8 S rRNA end formation has been described [12-14].

In previous studies, the human immune response targeting the PM/Scl complex has been reported to be predominantly directed against two polypeptides with apparent molecular masses of 100 kDa and 75 kDa [15]. In the past it has been shown that nearly all PM/Scl-positive sera contain autoantibodies to the 100 kDa protein and that only about 50–60% react with the 75 kDa protein [7,8,15-17]. A more recent study has shown that the PM/Scl-75 protein contains a previously unidentified N-terminal region that is important for the antigenicity of the protein [18]. The reactivity of sera with this new isoform of PM/Scl-75c is similar to the conventional PM/Scl-100 protein [18]. Several other components of the human exosome, including hRrp4p, hRrp40p, hRrp41, hRrp42p, hRrp46p and hCsl4p, are also recognized by anti-PM/Scl antibodies, but to a lesser extent [10,19].

In several studies during the past decade, we and others have attempted to identify the epitopes on PM/Scl-100 that are recognized by the cognate autoantibodies [12,20-23]. The prime reactivity of anti-PM/Scl-100 sera was localized to a domain of the protein represented by amino acids 231–245 using membrane-bound peptide arrays [22,23]. The amino acids contributing to the antibody binding were identified by mutational analysis [22,23]. Based on these observations and on secondary structure predictions, a local alpha-helical structure has been proposed for this major PM/Scl-100 epitope [22,23].

The aim of this study was to develop an ELISA with a 15-mer peptide comprising the PM/Scl-100 major epitope as a substrate, and to evaluate its sensitivity and specificity for the detection of anti-PM/Scl antibodies.

## Materials and methods

### Serum samples

In the present study three different serum panels were used to analyze the accuracy of the alpha helical PM/Scl-100 epitope (PM1-α) peptide in the ELISA. For the technical comparative study, 33 sera with anti-PM/Scl reactivity were preselected by indirect immunofluorescence on HEp-2 cells and cryopreserved monkey liver sections (Euroimmun, Lübeck, Germany) and by immunoblot with total cell extracts (Panel I). Panel II consisted of sera from a previous study and included patients with PM/Scl, patients with PM, patients with Scl, patients with dermatomyositis (DM) patients with melanoma and normal donors [18]. For the multicenter evaluation, serum samples were collected from patients with PM/Scl overlap syndrome (*n *= 40), from patients with Scl (*n *= 50), from patients with PM (*n *= 40) and from patients with various control diseases including rheumatoid arthritis (*n *= 69), SLE (*n *= 114), undifferentiated connective tissue disease (*n *= 10), mixed connective tissue disease (*n *= 6), Hashimoto thyroiditis (*n *= 11), Grave's disease (*n *= 12), other autoimmune disorders (n = 8), and hepatitis C virus infection (HCV) (*n *= 48) (Panel III).

PM/Scl patients were diagnosed based on the official PM and Scl criteria and were only considered true overlap patients if they fulfilled both the criteria for PM and for Scl [24,25]. All other patients with autoimmune disorders were classified according to the official criteria for each disease as also applied in a recent investigation [26]. Sera were stored in aliquots at -80°C until use and were shipped on dry ice. Collection of patient samples was carried out according to local ethics committee regulations.

### Antigens for ELISA

The identified sequence LDVPPALADFIHQQR of the PM/Scl-100 (accession number JH0796) major B-cell epitope covering amino acids 231–245 was used to synthesize the PM1-α peptide with an additional cysteine residue at the C-terminus using Fmoc chemistry [22]. Crude peptide obtained from peptide synthesis was purified by high-performance liquid chromatography. The quality and purity of the peptide was assessed by mass spectrometry and analytical high-performance liquid chromatography. The molecular mass was found at 1824.1167 Da (average; monoisotopic mass = 1822.9274 Da) and a purity of 100% was determined. The isoelectric point of the peptide was 4.0. Recombinant PM/Scl-100 (Diarect AG, Freiburg, Germany), was expressed in *Escherichia coli *and purified via a His-tag, and the quality was ensured by immunoblot and checkerboard analysis of positive and negative sera in the ELISA [27].

### Indirect immunofluorescence

Indirect immunofluorescence (IIF) was carried out using BioChip-mosaics with HEp-2 cells and primate liver as substrates (lot number 10116D; Euroimmun GmbH). Antibody titers were determined using 10-fold serial dilutions in PBS and the assay was performed according the manufacturer's instructions.

### Immunoblotting

Total cell extracts from HEp-2 cells that were separated by SDS-PAGE and transferred onto nitrocellulose were used as substrate for immunoblotting (lot numbers 01011a-88 and 01011a-89; Euroimmun GmbH). The identity of the PM/Scl antigens was ensured using PM/Scl index sera, which were previously characterized by several methods. Sera were diluted and incubated according to the manufacturer's instruction.

### ELISA

The PM1-α peptide was absorbed onto 96-well polystyrene plates (maxisorb; Nunc, Rosilke, Denmark) by overnight incubation at 4°C in 0.1 M carbonate buffer (pH 9.5). Different coating concentrations and different blocking, washing and incubation conditions were compared to optimize the assay conditions. Finally, the evaluation of antibody binding to the PM1-α peptide was performed as follows. Serum samples diluted 1:100 in dilution buffer at a volume of 100 μl/well were incubated for 30 min. After washing three times with washing buffer, anti-human IgG conjugate was added to the wells (100 μl/well) and incubated for 30 min. Surplus conjugate was removed by three washing cycles. The substrate was finally added to each well (100 μl/well) and incubated for 15 min. After stopping the color reaction with stop solution, the absorbance was measured at 450 nm. All steps were carried out at room temperature.

A highly positive index patient serum that was available in larger quantities was used to generate a calibrator. The sample was diluted 1:200 to yield an optical density of about 2.0 in the ELISA. The optical density of each patient sample was divided by the optical density of the calibrator and the result was multiplied by 10. For the technical comparison, the cut-off value of the prototype kits was based on the mean ± three standard deviations of 12 healthy blood donors. During the multicenter study the cut-off was validated and optimized by receiver operating characteristic analysis (see later).

All ELISAs using recombinant proteins were performed as already described, using recombinant proteins expressed in *E. coli *and purified using either a His-tag or ion-exchange chromatography [17,18].

### Addressable laser bead immunoassay

Microspheres embedded with laser reactive dyes (Luminex Corporation, Austin, TX, USA) that were coupled with autoantigens were part of a commercial kit (QUANTA Plex 8 TM; INOVA Diagnostics Inc., San Diego, CA, USA). This profile test allows for the semiquantitative detection of autoantibodies to chromatin, Jo-1, Rib-P, RNP, Scl-70, Sm, SS-A (Ro) and SS-B (La). The assay was performed according to the manufacturer's instructions. Briefly, each test serum was diluted to 1/1000 and 50 μl was added to a well of a microtiter plate, mixed with the antigen-coated beads that were preserved in the well, and incubated for 30 min. Then 50 μl phycoerythrin-conjugated goat anti-human IgG (Jackson ImmunoResearch Laboratories Inc., West Grove, PA, USA) was added to each well and incubated for an additional 30 min. The reactivity of the antigen-coated beads was determined on a Luminex 100™ dual laser flow cytometer (Luminex Corporation). The cut-off for a positive test result was based on the reactivity of control samples. The control samples were titrated to provide high, medium, low and negative values. Further information is available online .

### Statistical evaluation of the results

The results obtained from the comparative study were evaluated using Analyse-it software (Version 1.62; Analyse-it Software, Ltd, Leeds, UK). Receiver operating characteristic curves, positive predictive values and negative predictive values, as well as the test efficiency, were calculated. Furthermore, the correlation coefficients between the immunoassays based on the different antigens were calculated.

## Results

### Technical comparison of IIF, immunoblot and ELISA for the detection of anti-PM/Scl antibodies

To compare the different techniques, 33 anti-PM/Scl sera preselected on the basis of their IIF pattern and/or immunoblot result were tested in prototype ELISA kits based on the full-length recombinant PM/Scl-100 polypeptide expressed in *E. coli *and on the synthetic PM1-α peptide. In total, 26/33 (78.8%) were positive in the ELISA with the recombinant protein and 32/33 (97.0%) were positive in the ELISA with the synthetic peptide. Results are summarized in Table 1. Based on the high sensitivity of the peptide-based ELISA in this technical comparison, we evaluated the clinical accuracy of the assay in an extended multicenter study using clinically defined sera from various centers.

### Correlation of anti-PM1-α with anti-PM/Scl-75a, PM/Scl-75c and PM/Scl-100 reactivity in ELISA

A panel of sera (*n *= 81) tested previously for reactivity to recombinant PM/Scl proteins (Panel II) was assayed for anti-PM1-α peptide reactivity in the ELISA. The results were compared with the known reactivity of these sera with the recombinant proteins [18]. When all assays were adjusted to the same specificity (91.1%), the clinical sensitivity for the PM/Scl overlap syndrome was 36.1% for PM1-α, was 27.8% for PM/Scl-75c and was 25.0% for PM/Scl-100.

**Figure 1:**
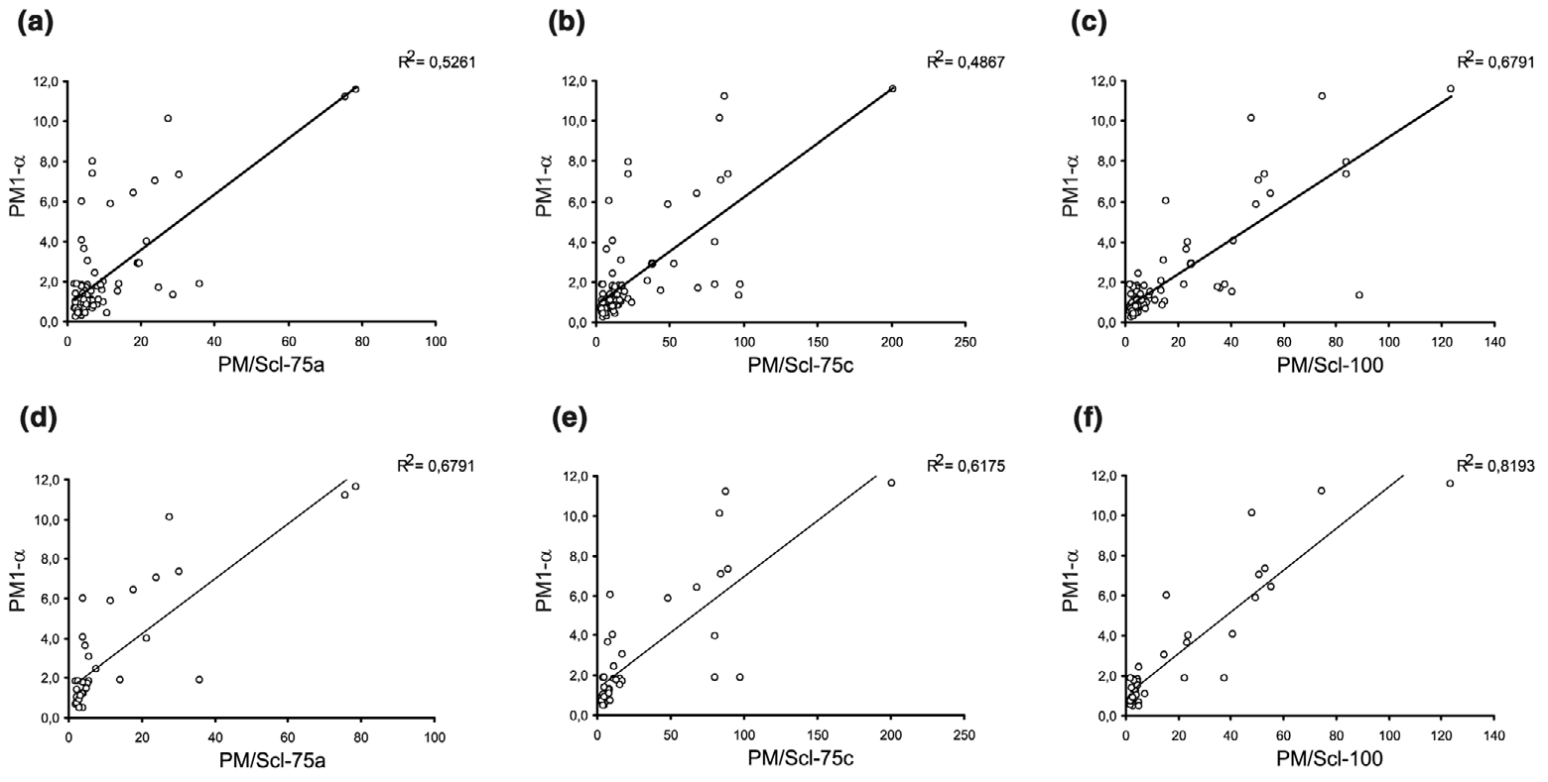
Correlation diagrams of PM1-α, PM/Scl-75a, PM/Scl-75c and PM/Scl-100. A panel of sera tested previously for reactivity to recombinant polymyositis/scleroderma (PM/Scl) components (PM/Scl-75a, PM/Scl-75c and PM/Scl-100) was assayed for anti-PM1-α peptide reactivity in an ELISA [18]. Correlation diagrams are shown comparing the peptide ELISA with the recombinant proteins **(a)–(c) **for all sera (*n *= 81) and **(b)–(f) **for only the sera of PM/Scl patients (*n *= 36).

There was a clear correlation between the peptide reactivity and the reactivity of the sera with the recombinant proteins. Not surprisingly, the strongest correlation was observed with the anti-PM/Scl-100 reactivity (Fig. 1). Whereas the majority of the sera showed comparable reactivity in all four assays, some individual samples showed a higher reactivity to the recombinant proteins than to the synthetic peptide, and vice versa. Overall, only one sample (from a patient with DM) was found that tested positive for the recombinant proteins but negative for the synthetic peptide. However, 11 sera that tested positive in the peptide ELISA remained undetected using the recombinant proteins.

When analyzing only the PM/Scl patients from this panel (36/81), the correlation between the reactivity of the peptide and the recombinant proteins was even higher (PM/Scl-100, *R*^2 ^= 0.82). Very importantly, no sera were found positive for PM/Scl-75c and/or PM/Scl-100 but negative for the PM/Scl peptide in the PM/Scl patient group. Two samples were PM/Scl-75c-positive (new isoform), PM1-α-positive and PM/Scl-100-negative. One sample reacted with PM/Scl-100 and PM1-α but not with the PM/Scl-75 proteins. Of the PM/Scl sera, 27.8% (10/36) was positive for the peptide but was negative for all recombinant polypeptides.

### Correlation with other autoantibodies

A statistical evaluation was performed using a patient cohort of 70 clinically defined PM/Scl sera and PM sera to evaluate correlations between anti-PM1-α peptide antibodies and other autoantibodies in ELISA assays using recombinant proteins. No significant correlation was found with Ro-52, Ro-60, La or Mi-2 antibodies (Table 2). Anti-PM1-α antibodies and anti-Jo-1 reactivity were negatively correlated. In addition, a reduced number of samples from 28 patients with clinically defined PM/Scl were also tested in an addressable laser bead immunoassay for autoantibodies to chromatin, Rib-P, RNP, Scl-70 and Sm, SS-A (Ro) and SS-B (La) (QUANTA Plex 8 TM; INOVA Diagnostics Inc.). Although antibodies to chromatin, Rib-P and RNP were detected in some patients, none of these antibodies appeared to be coincident with anti-PM1-α reactivity (Table 2).

### Multicenter evaluation of the PM1-α ELISA

**Figure 2:**
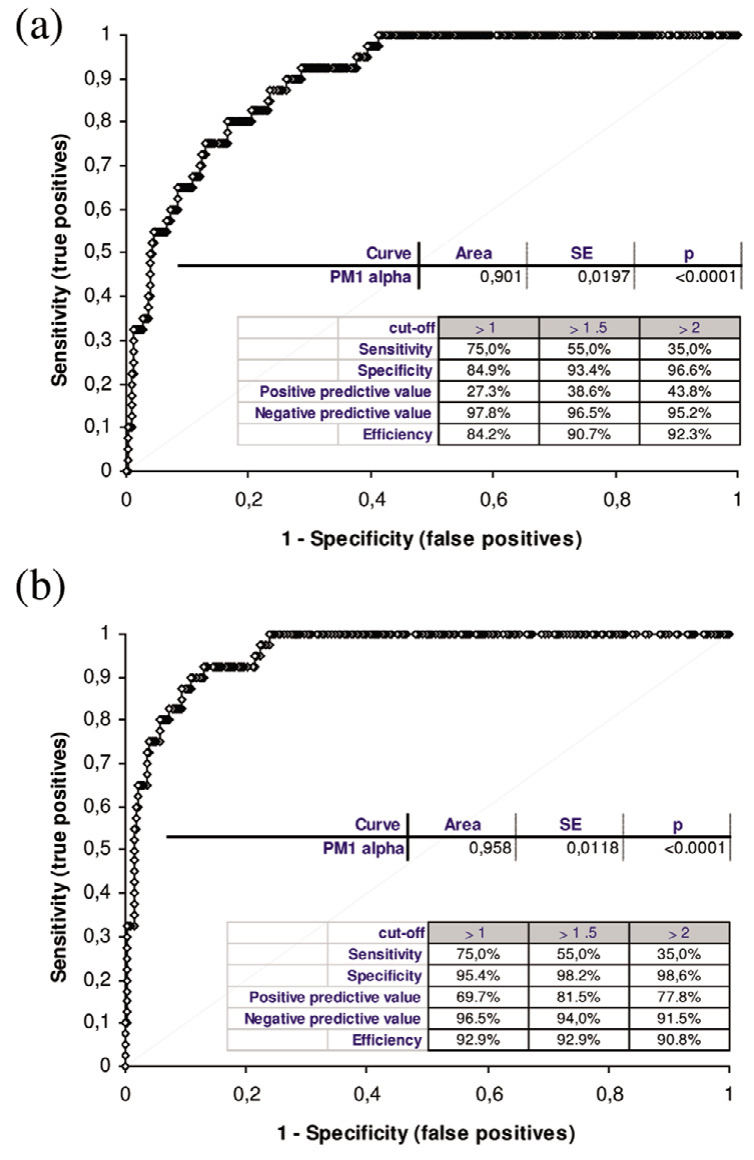
Receiver operating characteristic analysis of the PM1-α ELISA. Results obtained from three centers and based on 567 patients including polymyositis/scleroderma (PM/Scl) patients (*n *= 40), Scl patients (*n *= 205) and PM patients (*n *= 40) as well as other controls were used to calculate a receiver operating characteristic analysis **(a) **for all control samples and **(b) **for unrelated controls (without Scl and PM). The curve shows a clear discrimination between PM/Scl patient samples and various controls as emphasized by an area under the curve value of 0.901 (all controls) and 0.958 (unrelated controls). The differentiation between PM/Scl patients and controls was significantly improved when Scl patients and PM patients were excluded from the control group (b). SE, standard error.

**Figure 3:**
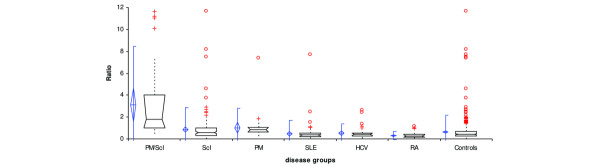
Reactivity of polymyositis/scleroderma (PM/Scl) patients and controls in the PM1-α ELISA. Results obtained from three centers and based on 567 patients including PM/Scl patients (*n *= 40), Scl patients (*n *= 205) and PM patients (*n *= 40) as well as other controls were used to calculate comparative descriptive analysis. The diagram shows a significantly increased reactivity of the PM/Scl sera compared with the control groups. Comparative descriptives show vertical box-plots for each sample, side by side for comparison. The blue line series shows parametric statistics: diamond, mean and the requested confidence interval around the mean; notched line, requested parametric percentile range. The notched box and whiskers show non-parametric statistics: notched box, median, lower and upper quartiles, and confidence interval around the median; dotted line, connects the nearest observations within 1.5 interquartile ranges (IQR) of the lower and upper quartiles. + and ○, possible outliers – observations more than 1.5 IQR (near outliers) and more than 3.0 IQR (far outliers) from the quartiles. Vertical lines, requested nonparametric percentile range. SLE, systemic lupus erythematosus; HCV, hepatitis C virus; RA, rheumatoid arthritis.

Sera from 40 clinically defined but serologically unselected patients with PM/Scl overlap syndrome, as well as from 205 Scl patients, 40 PM patients and various other controls (Panel III), were analyzed in the PM/Scl peptide ELISA (see Table 3). The results from all patients were used to calculate a receiver operating characteristic curve, which showed a clear discrimination between PM/Scl patients and various controls (Fig. 2). At a selected cut-off value of 1.5 RU, 22/40 (55%) PM/Scl patients tested positive for anti-PM1-α antibodies displaying a reactivity of up to 11.6 RU with a mean value of 3.1 ± 3.2 RU (Table 3). Patients from related disorders including Scl and PM showed a lower mean reactivity compared with the overlap patients but a higher reactivity than more unrelated controls. In total, 27/205 (13.2%) scleroderma patients (mean 0.7 ± 1.3 RU) and 3/40 (7.5%) PM patients (mean 1.0 ± 1.1 RU) tested positive, while 3/114 (2.6%) patients with SLE and 2/48 (4.2%) patients with HCV infection had anti-PM1-α antibodies. None of the remaining controls showed reactivity to the PM1-α peptide in the ELISA (Table 3, Fig. 3).

In total, 6.6% control sera tested positive for anti-PM1-α antibodies. This resulted in a diagnostic sensitivity of 55% and a specificity of 93.4% of the peptide ELISA (positive predictive value = 38.6%, negative predictive value = 96.5%, test efficiency = 90.7%). When Scl patients and PM patients were excluded from the group of controls 5/288 (1.7%) patients were positive, resulting in a specificity of 98.2% (positive predictive value = 81.5%, negative predictive value = 94.0%, test efficiency = 92.9%). These data indicate that, within the assay parameters used here, anti-PM1-α antibodies appear to be mainly present in sera from PM/Scl patients, from Scl patients, and to a lesser extent, PM patients.

## Discussion

The aim of this study was to compare the autoantigenicity of the PM1-α peptide that we have described previously [22,23] with that of native and recombinant PM/Scl-75 and PM/Scl-100 polypeptides. The results of the technical comparison showed that the PM1-α peptide ELISA is more sensitive than the ELISA tests based on the recombinant proteins, and than immunoblot and IIF experiments. Also, our results suggest that increased titers of autoantibodies directed to PM1-α might be more prevalent in patients with the PM/Scl overlap syndrome and related diseases than autoantibodies to the full-length proteins, which up to now were considered the most frequently present.

In the past, the presence of these antibodies in serum was generally monitored by IIF with HEp-2 cells, by immunodiffusion assays with calf thymus extract and/or by immunoblot using extractable nuclear antigens [4,5,15]. All these techniques allow the detection of a wide variety of autoantibodies present in patient serum [2]. The detection of anti-PM/Scl antibodies by immunoblotting, however, is difficult, because the reactivity of the antibodies with particularly PM/Scl-75 in cell extracts is notoriously weak in immunoblot, which may be due to the importance of conformational epitopes [15]. This observation could be confirmed in the technical comparison of IIF, immunoblot and ELISA in the present study. In recent years, ELISA using recombinant PM/Scl-100 has become a common method to detect anti-PM/Scl reactivity because it can easily be applied in an automated setting.

Since anti-PM/Scl-75 reactivity was previously detected only in patient sera that also contained anti-PM/Scl-100 autoantibodies [15], this protein is usually not included in such assays. A recent investigation has shown that also the use of an incomplete recombinant PM/Scl-75 polypeptide may have led to an underestimation of the diagnostic value of the PM/Scl-75 antigen [18].

We recently characterized the antibody response to a major PM/Scl epitope and found that 14/14 (100%) samples with PM/Scl antibodies demonstrated reactivity to the major epitope in a membrane-based peptide array [22,23]. We have characterized the major PM/Scl-100 B-cell epitope at the amino acid level and identified the key amino acids involved in antibody binding [22,23]. Using this peptide as an antigen, we developed a highly sensitive and specific ELISA system that detects a subpopulation of anti-PM/Scl antibodies present in 55% of PM/Scl patients, in 13.2% of Scl patients and in 7.5% of PM patients. Interestingly, this peptide also contains a generalized T-cell epitope pattern (ALADFIHQQR; amino acids 236–245) as well as several major histocompatibility complex epitopes [28-30].

Synthetic peptides represent ideal antigenic targets for immunoassays because they can easily be produced in high quality and quantity. Furthermore, less lot-to-lot variation will be observed since the production is not dependent on the biological variation of native sources of antigens. More and more synthetic peptides are being used in immunological assay systems to detect autoantibodies. Some of them show higher specificities and sensitivities than the corresponding assay with recombinant protein or native protein as substrate [23].

The combined use of different PM/Scl antigens, including the recombinant PM/Scl-100 and the recently identified isoform of PM/Scl-75, as well as the PM1-α peptide, may represent the most sensitive and specific method to detect antibodies to the human exosome. Advances in multi-analyte technologies such as line assays, multiplex systems and micro-arrays allow for the development of sophisticated profile assays containing multiple different antigens. This may improve the diagnosis of a variety of disorders, especially of autoimmune diseases since for most of those disorders no highly sensitive marker is available. The diagnosis of PM/Scl, Scl and PM might be improved by providing an antigen array that includes different PM/Scl antigens in combination with Scl-70 (topoisomerase I), Ku70/86, centromere proteins, RNA polymerase, NOR-90, Jo-1, Mi-2, PL-7, PL-12 and fibrillarin.

Taken together, the use of the PM/Scl-100 synthetic peptide in an ELISA remarkably improves the clinical identification of patients with the PM/Scl overlap syndrome. Although the prevalence of autoantibodies recognizing most other exosome subunits is relatively low [8,10,11], the co-occurrence of antibodies targeting different exosome subunits in patient sera might be indicative of intermolecular epitope spreading and might be a marker for the overlap syndrome. The co-occurrence of anti-PM/Scl-100 and anti-PM/Scl-75 seems to be particularly associated with the PM/Scl overlap syndrome [18], but whether the use of even more components of the human exosome will further increase the sensitivity of these assays remains to be investigated.

Apart from patients with PM/Scl overlap syndrome and patients with Scl or PM alone, two HCV-positive and three SLE patients displayed reactivity to the PM1-α peptide in ELISA. HCV infection has been associated with a plethora of immune and autoimmune perturbations [31]. Although the cause and effect remain to be proved, there are reports of HCV infection preceding or coincident with polyarthritis, rheumatoid arthritis, SLE, and PM/DM. The role of anti-PM1-α antibodies in HCV patients and SLE patients remain a matter for further investigation. In the present study we found antibodies to the PM1-α peptide present in 13.2% of unselected scleroderma patients. In only a few of those patients was a history of myositis documented. It is possible that the myositis was mild in the majority of patients and was completely overlooked by the examining clinician or that the antibody precedes the associated clinical features [32]. We therefore conclude that the complete autoantibody profile is important for a careful examination of patients with rheumatic diseases and to access all their clinical features.

Frank and colleagues analyzed sera from 216 patients with idiopathic inflammatory myopathies to assess putative associations between anti-SS-A/Ro-52 and other autoantibodies. These included sera containing antibodies that recognize Jo-1, Mi-2, PM/Scl, signal recognition particle, as well as the scleroderma-related antibodies anti-topoisomerase I (Scl-70) and anti-centromere. A high proportion of sera that contain anti-Jo-1 antibodies, anti-signal recognition particle or anti-PM/Scl antibodies were found to contain antibodies to the Ro52 protein [33]. The reported association between anti-Ro-52 and anti-PM/Scl antibodies is not found in our cohort. Although our correlation study is based on a limited number of samples, we found no correlation between anti-PM/Scl antibodies and anti-Ro52. In contrast, Yamanishi and colleagues reported an association of PM/Scl syndrome with anti-Ku antibody and rimmed vacuole formation [34]. Similar to this observation, we found that two out of 29 (6.9%) anti-PM/Scl-positive samples also were positive for anti-Ku86 antibodies. In a previous study it became evident that the PM/Scl-100 major epitope shares some sequence homology to an amino acid stretch (amino acids 58–72) of the heterochromatin protein p25β, which is frequently the target of anti-chromo antibodies from a subpopulation of patients also having anti-centromere antibodies [22]. Although none of 14 PM1-α-positive samples showed reactivity to the corresponding region of p25β, a more complex immunological relationship between the major PM/Scl-100 epitope and the corresponding p25β peptide cannot be excluded. More samples with anti-PM/Scl and anti-chromo antibodies have to be tested for cross-reactivity. Further studies are required to analyze the association of anti-PM1-α antibodies with other known autoantibodies.

Today's sophisticated epitope mapping methods will probably lead to the identification of additional peptides, which can be used as specific targets in diagnostic and therapeutic approaches to patient management. This may lead to a new scientific research area with high impact for the development of diagnostic and therapeutic products – to the area of peptide engineering.

## Conclusion

In the present study, we showed that the detection of anti-PM/Scl antibodies using an ELISA system based on a major PM/Scl-100 epitope is remarkably improved compared with conventional detection methods. It could be shown that a subpopulation of PM/Scl antibodies directed against the PM1-α peptide is present in 55% of PM/Scl patients, in 13.2% of Scl patients and in 7.5% of PM patients. In rare cases anti-PM1-α reactivity was also found in patients suffering from HCV, SLE or melanoma. Within our patient cohorts we found no statistical evidence of a positive association between anti-PM1-α and antibodies other than to PM/Scl components. Based on the results of the present study we conclude that anti-PM1-α antibodies are exclusively present in sera from patients suffering from Scl or PM and most frequently in patients with the PM/Scl overlap syndrome. We therefore conclude that the new anti-PM1-α ELISA test offers a new serological test that will improve the diagnosis of complex connective tissue disorders.

## Abbreviations

DM = dermatomyositis; ELISA = enzyme-linked immunosorbent assay; HCV = hepatitis C virus; IIF = indirect immunofluorescence; PBS = phosphate-buffered saline; PM = polymyositis; PM1-α = alpha helical PM/Scl-100 epitope; RU = relative units; Scl = scleroderma; SLE = systemic lupus erythematosus.

## Competing interests

MM is employed at Dr Fooke Laboratorien GmbH, which may commercialize the assay.

## Authors' contributions

MM developed and validated the ELISA system, planned the experiments and filed the manuscript. MJF and RR delivered clinically defined sera, advised MM in evaluating the clinical part of this study and contributed to the preparation of the manuscript. CD organized the analysis of anti-PM/Scl samples in IIF and immunoblot. MB advised MM during the characterization of the PM1-α peptide.

## Figures and Tables

**Table 1:** Results of the technical comparison of indirect immunofluorescence (IIF), immunoblot and ELISA for the detection of anti-polymyositis/scleroderma (anti-PM/Scl) antibodies

Sample number	IIF		Immunoblot			ELISA	
	
	Titer	Pattern	PM/Scl-75	PM/Scl-100	Other	PM/Scl-100	PM1-α
1	1:10000	FG, N	++	+++		15.3	17.7
2	1:1000	FG, N	+	+		2.9	7.6
3	1:3200	FG, N	++	++		14.4	29.2
	1:320	AMA					
4	1:320	FG, N	-	+		1.1	4.7
5	1:1000	FG, N	+	+		2.2	12.1
6	1:3200	FG, N	-	-		0.6	2.3
7	1:1000	FG, N	++	+++		5.2	13.3
8	1:1000	FG, N	+	+		2.3	12.7
9	1:3200	FG, N	+	+++	P38	15.5	36.1
10	-		-	+		1.7	4.0
11	1:1000	FG, N	-	++		19.2	2.4
12	1:1000	FG, N	++	+		1.2	3.2
13	1:3200	FG, N	+++	+++	Ku86, Jo-1	6.5	10.6
14	1:3200	FG, N	-	++		2.1	5.9
15	1:3200	FG, N	++	+++		11.4	22.5
16	1:1000	FG, N	-	+		3.6	8.5
17	1:1000	FG, N	+	++		3.2	15.0
18	1:3200	FG, N	++	++		6.1	11.8
19	n.d.	n.d.	-	++		7.1	19.4
20	1:320	N	-	+		2.1	5.6
21	n.d.	n.d.	+++	++		4.1	12.8
22	1:10000	FG	-	+	P38	0.7	1.5
	1:10000	SPA					
23	1:1000	N	n.d.	n.d.		0.5	2.7
24	n.d.	n.d.	++	+++		18.2	34.7
25	1:10000	N	n.d.	n.d.	P38	0.5	2.0
	1:1000	Rib					
26	1:1000	N	n.d.	n.d.		1.9	8.4
27	n.d.	n.d.	+	++		5.9	19.4
28	n.d.	n.d.	++	+++		20.9	37.3
29	1:1000	Hom	n.d.	n.d.	Ku86, Cen, M2	0.6	0.8
	1:100	N*					
30	1:1000	N	-	+		0.6	1.4
31	1:3200	Hom, N	-	-		0.5	2.9
32	1:3200	N	-	-		0.5	1.3
33	1:320	FG, N	+	++		7.2	25.7
Number positive/tested		26/29	17/29	27/29		26/33	32/33

-, negative; +, weak positive; ++, positive; +++, strong positive; n.d., not determined; FG, fine granular; Hom, homogenous; SPA, spindle apparatus; N, nucleoli; AMA, anti-mitochondrial antibodies; Cen, centromere; Rib, ribosomal. * Primate liver.

**Table 2:** Correlation of anti-PM1-α and other known autoantibodies

	Number positive/all sera (% positive)	Number positive/PM1-α-positives (% positive)	Number positive/PM1-α-negatives (% positive)	*P*
Scl-70	0/28 (0)	0/15 (0)	0/13 (0)	*
Sm	0/28 (0)	0/15 (0)	0/13 (0)	*
Rib-P	1/28 (3.6)	1/15 (6.7)	0/13 (0)	*
RNP	4/28 (14.4)	1/15 (6.7)	3/13 (23.1)	*
Chromatin	1/28 (3.6)	1/15 (6.7)	0/13 (0%)	*
Ro-52	18/70 (25.7)	6/19 (31.6)	12/51 (23.5)	0.7056
Ro-60	6/70 (8.6)	2/19 (10.6)	4/51 (7.8)	0.9018
La	3/70 (4.3)	1/19 (5.3)	2/51 (3.9)	0.6766
Mi-2	10/70 (14.3)	3/19 (15.8)	7/51 (13.7)	0.8693
Jo-1	16/70 (22.9)	1/19 (5.2)	15/51 (29.4)	0.0688

* Not calculated due to the limited number of samples.

**Table 3:** Results of ELISA using PM1-α peptide with polymyositis/scleroderma and various control sera

	Number (%) of anti-PM1-α-positive sera	Mean value/standard deviation	Top value
Polymyositis/scleroderma (*n *= 40)	22 (55)	3.1/3.2	11.6
Rheumatic disease controls (*n *= 452)	33 (7.3)	0.6/0.9	7.7
Polymyositis (*n *= 40)	3 (7.5)	1.0/1.1	7.4
Scleroderma (*n *= 205)	27 (13.2)	0.9/1.2	7.5
Rheumatoid arthritis (*n *= 69)	0 (0)	0.3/0.2	1.1
Mixed connective tissue disease (*n *= 6)	0 (0)	0.4/0.1	0.6
Undifferentiated connective tissue disease (*n *= 10)	0 (0)	0.3/0.0	0.4
Systemic lupus erythematosus (*n *= 114)	3 (2.6)	0.5/0.7	7.7
Other rheumatic diseases (*n *= 8)	0 (0)	0.3/0.1	0.6
Hepatitis C virus (*n *= 48)	2 (4.2)	0.5/0.5	2.6
Organ specific disorders (*n *= 23)	0 (0)	0.4/0.2	0.8
Hashimoto thyroiditis (*n *= 11)	0 (0)	0.3/0.2	0.8
Grave's disease (*n *= 12)	0 (0)	0.4/0.2	0.8
Healthy individuals (*n *= 4)	0 (0)	0.6/0.2	0.7
